# Sport-Related Concussion and Mental Health Outcomes in Elite Athletes: A Systematic Review

**DOI:** 10.1007/s40279-017-0810-3

**Published:** 2017-11-20

**Authors:** Simon M. Rice, Alexandra G. Parker, Simon Rosenbaum, Alan Bailey, Daveena Mawren, Rosemary Purcell

**Affiliations:** 1 Orygen, The National Centre of Excellence in Youth Mental Health, 35 Poplar Road, Parkville, Melbourne, VIC 3052 Australia; 2 Centre for Youth Mental Health, The University of Melbourne, Melbourne, VIC Australia; 3 Youth Mood Clinic, Orygen Youth Health, Melbourne, VIC Australia; 4 Institute of Sport, Exercise and Active Living, Victoria University, Melbourne, VIC Australia; 5 School of Psychiatry, University of New South Wales, Sydney, NSW Australia; 6 Black Dog Institute, Prince of Wales Hospital, Sydney, NSW Australia

## Abstract

**Background:**

Elite athletes can experience a diverse range of symptoms following post-concussive injury. The impact of sport-related concussion on specific mental health outcomes is unclear in this population.

**Objective:**

The aim was to appraise the evidence base regarding the association between sport-related concussion and mental health outcomes in athletes competing at elite and professional levels.

**Methods:**

A systematic search of PubMed, EMBASE, SPORTDiscus, PsycINFO, Cochrane, and Cinahl databases was conducted.

**Results:**

A total of 27 studies met inclusion criteria for review. Most of the included studies (67%, *n* = 18) were published in 2014 or later. Study methodology and reporting varied markedly. The extant research has been conducted predominantly in North America (USA, *n* = 23 studies; Canada, *n* = 3), often in male only (44.4%, *n* = 12) and college (70.4%, *n* = 19) samples. Depression is the most commonly studied mental health outcome (70.4%, *n* = 19 studies). Cross-sectional retrospective studies and studies including a control comparison tend to support an association between concussion exposure and depression symptoms, although several studies report that these symptoms resolved in the medium term (i.e. 1 month) post-concussion. Evidence for anxiety is mixed. There are insufficient studies to draw conclusions for other mental health domains.

**Conclusion:**

Consistent with current recommendations to assess mood disturbance in post-concussive examinations, current evidence suggests a link between sports-related concussion and depression symptoms in elite athletes. Causation cannot be determined at this stage of enquiry because of the lack of well-designed, prospective studies. More research is required that considers a range of mental health outcomes in diverse samples of elite athletes/sports.

**Electronic supplementary material:**

The online version of this article (10.1007/s40279-017-0810-3) contains supplementary material, which is available to authorized users.

## Key Points

**Table:** 

We conducted a comprehensive systematic appraisal of the evidence regarding an association between sport-related concussion and mental health outcomes in elite athletes. Of the 27 studies included, most reported on depression, with a minority reporting outcomes related to anxiety, attention deficit hyperactivity disorder and impulsivity.
Small, prospective studies suggested acute/subacute effects for mood/depression symptoms that tend to resolve around 4 weeks post-concussion. Larger scale cross-sectional studies suggest a higher prevalence of depression symptoms associated with the retrospective recall of lifetime concussion(s).
Definitive conclusions about a relationship between concussion and mental health outcomes are limited given that 40.7% of included studies were deemed to have a possible risk of bias. Well-designed representative, prospective studies including a pre-exposure baseline assessment and multiple-time-point, post-concussion assessments are urgently needed.

## Introduction

Sport-related concussion is defined as the immediate and typically transient symptoms of traumatic brain injury induced by biomechanical forces [1, 2]. A range of neurological symptoms may result, largely reflecting functional rather than structural brain disturbance [3]. Post-concussive injury can occur across physical, cognitive and emotional domains [4], and there is growing interest in medical surveillance of athletes post-concussion [5]. A small proportion of athletes continue to experience persistent concussion symptoms, which may be precipitated or perpetuated by the severity of exposure (i.e. loss of consciousness or concussive convulsions), early post-traumatic or retrograde amnesia, comorbidities or pre-morbidities (i.e. migraine, mental health disorder) or younger age [6, 7].

Media reporting of high-profile athlete suicides or significant mood and behavioural disturbance has drawn public attention to the link between (repeated) concussive injuries and poor psychosocial outcomes later in life [8]. The potential negative psychological outcomes of sport-related concussion have certainly been considered within acute post-concussion assessment tools [9], and the current iteration of the Sport Concussion Assessment Tool (SCAT-5) [10] includes a number of psychological domains, such as problems with irritability, sadness, concentration, or feeling ‘more emotional’. The 2017 Concussion in Sport Group Consensus Statement also highlights the importance of post-concussive mental health symptoms, and advises that the development of depression post-concussion or the existence of pre-injury depression are likely risk factors for persistent (i.e. > 4 weeks) post-concussive symptoms [1]. Less is known, however, about the relationship between sport-related concussion and mental health outcomes *other* than depression, such as anxiety, substance misuse, psychosis/reality distortion, or personality disturbance.

Recent reviews of the association between concussion and mental health outcomes have been conducted with mixed samples of elite and non-elite, adult sport participants/athletes. These reviews have found concussion exposure to be a risk factor for subsequent mental health problems in some, but not all, individuals [11, 12]. Finkbeiner and colleagues’ review examined adult, sport-related concussion studies that assessed mental health at least 3 months post-concussive exposure [11]. The authors concluded that most studies suggested an increased prevalence of depressive symptoms related to concussion history, with inconsistent evidence for anxiety and substance use [11]. More recently, Manley and colleagues conducted a systematic review of sport-related concussion studies that reported long-term (i.e. > 10 year) outcomes across clinical, cognitive and brain imaging domains [12]. Manley and colleagues also found an association between later-life depression and multiple prior concussions (though not suicide), with a dose-response relationship identified between depression and concussion exposure in five studies [12]. No conclusions could be drawn, however, for other mental health outcomes. These reviews are highly informative from the perspective of characterising the range of potential outcomes that may be associated with sport-related concussion in a broad, mixed population; however, the specific association between concussion and mental health in elite and professional athletes is yet to be determined. This review conducted a broader, systematic search of the research literature to synthesise, appraise and interpret the current knowledge related to mental health outcomes and sport-related concussion in elite athletes. Focussing on this cohort is important given their elevated risk of exposure to high-impact contacts and concussion [13], and emerging data suggesting an association between concussion exposure and common mental disorders [14].

## Methods

### Literature Search

A systematic search of six electronic databases was conducted in December 2016 using relevant database search engines (PubMed, EMBASE, SPORTDiscus, PsycINFO, Cochrane, Cinahl). The search strategy and MeSH terms are presented in Table 1. Our search strategy was intentionally broad in order to be inclusive of studies that may not have been identified by previous reviews [11, 12].

**Table 1:** Search terms

Search #	Search terms	
#1	MeSH descriptor [brain concussion]	
#1	MeSH descriptor [concussion^a^]	
#2	MeSH descriptor [postconcussion^a^]	Title or Abstract^a^
#4	MeSH descriptor [brain injury]	
#5	MeSH descriptor [tbi]	
#6	MeSH descriptor [encephalopathy]	
#7	MeSH descriptor [head injury]	
#8	#1 or #2 or #3 or #4 or #5 or #6 or #7	
#9	MeSH descriptor [Sport]	Explode all trees
#10	Sport^a^	
#11	Player^a^	
#12	Athlete^a^	
#13	#9 or #10 or #11 or #12	

^a^All restricted to title/abstract and keyword

### Study Inclusion

Prior to screening by title and abstract, a random sample 500 articles were pre-screened by two members of the research team in order to ensure the study inclusion and exclusion criteria were clear. The two researchers met to discuss their results twice during this phase. Subsequent to this, a further random sample 100 articles were pre-screened by all five independent researchers. During this pre-screening stage the researchers met several times to discuss discrepancies. During this process, agreement was reached for the 100 articles, and the final wording of the screening inclusion and exclusion criteria was clarified. The five researchers then independently assessed the eligibility of the retrieved records on the basis of title and abstract. Records were equally divided amongst the team, based on alphabetised grouping of the first author surname. If title and abstract information was unclear (i.e. the researcher was unable to clearly determine whether the study met inclusion criteria at this first screen), the article was included for assessment in the full-text screening stage.

**Fig. 1:**
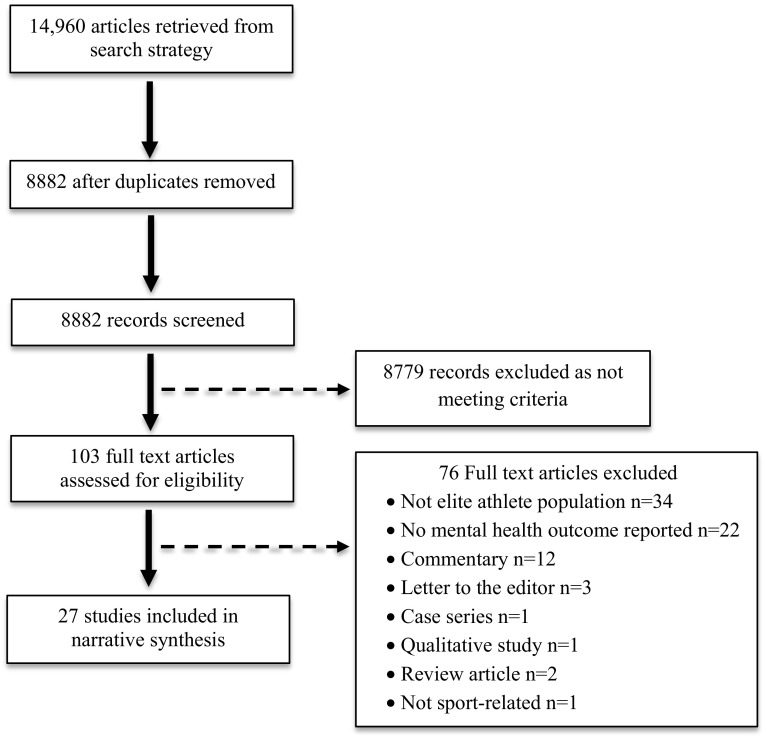
Study selection flow diagram

Included studies were required to meet the following inclusion criteria: (1) studies conducted with currently contracted or retired elite level athletes, where elite level was defined a priori to be either competitive at the Olympic or professional (paid) level (for state, national or international competition) or US collegiate level; (2) studies reported quantitative data on a mental health (including substance use), wellbeing, or coping outcome along with concussion; and (3) studies were published in a peer reviewed journal. Studies were excluded from the review based on the following criteria: (1) the study was qualitative or a case series, discussion paper, commentary or a literature review; (2) the study was conducted with a heterogeneous sample (i.e. a mixed sample of elite and non-elite athletes) without reporting group findings separately; (3) the study reported only on physiological wellbeing (including sleep), structural, cognitive or neuropsychological responses without assessing or reporting psychological wellbeing or mental health; (4) the study was available as an abstract only (i.e. conference presentations), precluding full quality assessment; (5) the study focussed on substance use in relation to performance enhancement (i.e. doping) as opposed to personal use; and (6) the study was unavailable in English. The systematic review was conducted in accordance with the Preferred Reporting Items for Systematic Reviews and Meta-Analyses (PRISMA) guidelines (see Fig. 1 for flow diagram).

### Data Extraction

A standardised data extraction template was designed. Six researchers sourced the required information from the included studies using this template, including study type and design, sport population, study aim, sex ratio, and key outcomes mapped to study measures and main findings.

## Results

### Literature Search

The literature search yielded a total of 14,960 records. An additional 485 articles were retrieved via soft searching. After removal of duplicates, screening of the titles and abstracts was conducted on 8882 articles. Of these, 103 studies were identified as likely meeting inclusion criteria and were subject to full review. Of these, 76 failed to meet inclusion criteria, leaving 27 studies included in the narrative review. Given the heterogeneity in study design and reporting of outcomes, it was not possible to conduct a meta-analysis.

### Description of Included Studies

Tables 2 and 3 summarise the key characteristics and main outcomes of the 27 included studies. For the purposes of reporting and analysis, studies were grouped according to major mental health constructs. In total, 37.0% (*n* = 10) of included studies reported on more than one mental health domain (i.e. anxiety and depression). Depression was the most frequently assessed mental health outcome, reported in 70.4% (*n* = 19) of the included studies, typically using self-report rating scales, including the Beck Depression Inventory 2 (BDI-II) (*n* = 7), Center for Epidemiologic Studies Depression Scale (CES-D) (*n* = 4), the Patient Health Questionnaire (PHQ-9) (*n* = 4) and the Hamilton Depression Rating Scale (*n* = 4). The next most frequently mentioned outcome was anxiety (25.9%, *n* = 7), followed by impulsivity (7.4%, *n* = 2) and attention deficit hyperactivity disorder (ADHD) (3.7%, *n* = 1). Five studies (18.5%) utilised multi-domain outcome measures. These studies used either the Profile of Mood States (POMS) (*n* = 3), the Patient-Reported Outcomes Measurement Information System (PROMIS) (*n* = 1), or the Veterans RAND 12-Item Health Survey (VR-12) (*n* = 1). Single studies reported on apathy (3.7%) and aggression (3.7%). Highlighting the rapid and growing interest in the field, most (67%) of the included studies were published in 2014 or later (i.e. ≤ 2012, *n* = 6; 2013, *n* = 3; 2014, *n* = 6; 2015, *n* = 6; 2016, *n* = 4, 2017, *n* = 2).

**Table 2:** Summary of included studies

Study	Year	Study type	Purpose	*N* ^a^ (M:F)	Mean age (SD), years	Sport	Location (country)	Sample
Banks et al. [15]	2014	Longitudinal, observational	To assess the brain health of fighters by use of cognitive assessment, neuroimaging, and self-reports of impulsiveness	131 (131:0)	28.5 (SD NR)	Mixed martial arts, boxing	Nevada (USA)	College and published control data
Casson et al. [16]	2014	Cohort	Assessment of the neuropsychological, clinical neurological and neuro-radiological symptoms of retired NFL players	45 (45:0)	45.6 (8.9)	American Football (NFL)	Various states (USA)	NFL players (retired)
Covassin et al. [17]	2014	Cross-sectional, comparative	Comparison of anxiety and social support of athletes with concussions vs a matched group of athletes with orthopaedic injuries	126 (92:34) (concussion exposed group, *n* = 63)	22.69 (SD 1.75)	Mixed (American Football, wrestling, softball, baseball, field hockey, soccer, basketball)	State not specified (USA)	College
Decq et al. [18]	2016	Cross-sectional, comparative	Comparison of exposure to recurrent concussion in sports and the incidence of depressive disorders, mild cognitive disorders, fluency disorders and headache	377 (377:0)	52 (Median)	Rugby	Various regions (France)	Rugby players (retired), elite sportsmen (retired)
Didehbani et al. [19]	2013	Cross-sectional, comparative	Investigation of depressive symptoms in retired professional American Football players with history of concussion	59 (59:0)	Exposed 58.6 (10.33); control 59.52 (10.64)	American Football (NFL)	Texas (USA)	NFL players with concussion history (retired), control group
Guskiewicz et al. [20]	2007	Cross-sectional	Investigation of the relationship between sport-related concussion and prevalence of lifetime clinical depression	2434 (2434:0)	53.8 (13.4)	American Football (NFL)	Various states (USA)	NFL players (retired)
Hart et al. [21]	2013	Cross-sectional	Assess for the presence of cognitive impairment and depression in aging former NFL players, and identify neuroimaging correlates of these dysfunctions in those with and without a history of concussion	34 (34:0)	61.8 (SD NR)	American Football (NFL)	Texas (USA)	NFL players (retired)
Hutchison et al. [22]	2009	Prospective cohort	Determine if athletes with concussion and those with minor musculoskeletal injuries experienced differential emotional responses post-injury	53 (33:20) (concussion exposed group 12:8)	20.05 (1.82)	Mixed (basketball, American Football, hockey, lacrosse, rugby, volleyball)	Canada	College
Kerr et al. [23]	2014	Cross-sectional	Examine current physical and mental health (including concussion history) in a cohort of former collegiate athletes who played in a diverse range of men’s and women’s sports	797 (376:421)	NR, ages ranged 22–51	Mixed (basketball, baseball, wrestling, equestrian)	Southern states (USA)	Former NCAA Division I in southern USA
Kerr et al. [24]^b^	2014	Cross-sectional	Estimate the association between recurrent concussion and current levels of depression, impulsivity, and aggression in a cohort of former collegiate athletes	797 (376:421)	NR, ages ranged 22–51	Mixed (basketball, baseball, wrestling, equestrian)	Southern states (USA)	Former NCAA Division I in southern USA
Kerr et al. [25]	2012	Prospective cohort	Prospectively determine the effects of recurrent concussions on the clinical diagnosis of depression in retired NFL players	1044 (1044:0)	63.1 (11.2)	American Football (NFL)	Various states (USA)	NFL players (retired)
Kontos et al. [26]	2012	Prospective cohort	Prospectively examine the relationship of sport-related concussion with depression and neurocognitive performance and symptoms among young athletes	21 (NR)	19.68 (1.33)	Mixed	State not specified (USA)	College
Mainwaring et al. [27]	2004	Prospective cohort	Determine the effects of mild traumatic brain injury on the longitudinal mood profiles of concussed college athletes in comparison to nonconcussed teammates and healthy comparisons	369 (231:138) (concussion exposed group 12:4)	21.17 (SD 2.94)	Mixed (basketball, American Football, hockey, lacrosse, mountain biking, rugby)	Toronto (Canada)	College
Mainwaring et al. [28]^c^	2011	Prospective cohort	Compare emotional response of athletes to concussion and to ACL injury	51 (21:30) (concussed exposed group 12:4)	21.2 (2.9)	Mixed (basketball, field hockey, American Football, hockey, lacrosse, mountain biking, rugby, soccer, volleyball)	Toronto (Canada)	College
Meehan et al. [29]	2016	Cross-sectional, retrospective	Determine whether the exposure to sub-concussive blows that occur during collision sports is associated with later-life neurobehavioral quality-of-life measures	3656 (2032:1624)	NR	Mixed (collision sports, contact sports, non-contact sports)	New England, (USA)	College
Meier et al. [30]	2015	Longitudinal and cross-sectional	Longitudinally assess recovery of CBF in collegiate athletes and compare time course of CBF recovery with that of cognitive and behavioural symptoms	44 (44:0) (concussed exposed group 17:0)	20.57 (1.2)	American Football	State not specified (USA)	College
Meier et al. [31]	2015	Prospective cohort	Examine patterns of symptoms reported in concussed athletes in different testing environments	40 (32:8)	20.44 (1.41)	Mixed (soccer, basketball, volleyball, football)	State not specified (USA)	College
Meier et al. [32]	2016	Longitudinal	To examine global and local connectivity in a sample of collegiate athletes at approximately 1 day, 1 week, and 1 month following sport-related concussion	94 (69:25) (concussed exposed group 34:9)	20.29 (1.31)	Mixed (basketball, American Football, rowing, soccer, volleyball)	State not specified (USA)	College
Montenigro et al. [33]	2016	Cross-sectional	Develop a metric to quantify cumulative RHI exposure from American Football, examine association between RHI exposure and clinical outcomes	76 (76:0)	47.7 (14.2)	American Football	State not specified (USA)	College
Poltavski and Biberdorf [34]	2014	Cross-sectional	Determine the utility of oculomotor-based evaluation protocols in screening for lifetime concussion incidence in elite hockey players	42 (21:21)	20.52 (NR)	Hockey	State not specified (USA)	College
Pryor et al. [35]	2016	Cross-sectional	Assess difference in concussive events among active professional and semi-professional American Football players with/without depression	27 (27:0)	26.6 (3.3)	American Football	State not specified (USA)	Professional/semi-professional
Putukian et al. [36]	2015	Prospective, cross-sectional	Evaluate whether the modifiers (age, sex, history of concussion, loss of consciousness) and depression and anxiety were associated with SCAT-2 concussion scores	263 (176:87)	20.33 (1.74)	Mixed (American Football, rugby volleyball)	State not specified (USA)	College
Roiger et al. [37]	2015	Prospective, case control	Examine post-injury depressive symptoms including post-concussion in a mixed collegiate sample	21 (NR) (7 concussed, 7 injured non-concussed, 7 healthy controls)	19.8 (1.4)	Mixed (wrestling, American Football, basketball)	State not specified (USA)	College
Singh et al. [38]	2015	Cross-sectional and longitudinal	Examine mood symptoms with kynurenine pathway metabolites following sport-related concussion	36 (36:0) (14 concussed in longitudinal component)	20.32 (1.07)	American Football	State not specified (USA)	College
Strain et al. [39]	2013	Cross-sectional	Examine relationship between white matter integrity (DTI imaging) and depression in retired NFL players	26 (26:0)	58.7 (11.9) symptomatic for depression; 54.0 (7.78) non-symptomatic	American Football (NFL)	Texas (USA)	NFL players (retired)
Vargas et al. [40]	2015	Prospective case control	Examine prevalence of depressive symptoms at baseline and post-concussion compared to controls	84 (65:19) (concussed exposed group 23:21)	18.4 (0.8) concussed; 18.9 (0.9) non-concussed	Mixed (American Football, lacrosse, basketball, soccer, ice hockey, wrestling)	State not specified (USA)	College
Yang et al. [41]	2015	Prospective	Examine the effect of baseline psychological symptoms on post-concussion symptoms	67 (NR)	NR	Mixed (American Football, basketball, baseball, soccer, hockey, volleyball, softball, wrestling)	State not specified (USA)	College

*ACL* anterior cruciate ligament, *CBF* cerebral blood flow, *DTI* diffusion tensor imaging, *F* female, *M* male, *NFL* National Football League, *NCAA* National Collegiate Athletic Association, *NR* not reported, *RHI* repetitive head impacts, *SCAT-2* Sport Concussion Assessment Tool-2, *SD* standard deviation

^a^Total *N* of sample after exclusion of high school athletes

^b^Same sample as Kerr et al. [23]

^c^Same sample as Mainwaring et al. [27]

**Table 3:** Summary of findings

Study	Concussion profile; *n* (%) reporting concussion, mean concussions	Mental health outcomes assessed (assessment tool)	Timing of mental health assessment relative to concussion	Main mental health findings
Banks et al. [15]	*n* = NR^a^; mean concussions for boxing = 0.4, range 0–11; mean concussions for mixed martial arts = 0.6, range 0–10	Impulsivity (BIS-15)	Self-reported lifetime concussions, sample did not experience recent concussion exposure	Athletes, compared to healthy male controls, had significantly lower total impulsiveness scores (60.5 vs 62.8; *p* = 0.012), lower self-control scores (11.5 vs 12.4; *p* = 0.032), and lower cognitive instability scores (5.3 vs 6.4; *p* < 0.001) and higher cognitive complexity scores (12.3 vs 11.3; *p* < 0.001). In MMA fighters, each increase of 10 years of fighting decreased attention scores by 1.5, *p* = 0.02
Casson et al. [16]	*n* = NR^a^; mean concussions = 6.9	Depression (BDI; PHQ-9)	Self-reported lifetime concussions, sample did not experience recent concussion exposure (retired athletes)	A total of 15 retired athletes (33%) reported any severity of depression; this was greater than the prevalence in the general population (i.e. 15–20%). Those with moderate-severe depression (*n* = 6; 13.3%) were consistent with general population rates. Similarly, 9 participants (20%) met PHQ criteria for depression, consistent with general population rates
Covassin et al. [17]	*n* = 63 (50%); mean concussions = NR	Anxiety (STAI)	Within 1 week of sustaining injury, either concussion or orthopaedic	Athletes in the concussion and orthopaedic injury groups reported equivalent state and trait anxiety, *p* = 0.193. The two groups were consistent in sources of social support sought during injury
Decq et al. [18]	*n* = 217 (68.5%)^a^; mean concussions for RPs = 3.1; mean concussions for OS = 0.68	Depression (PHQ-9)	Self-reported lifetime concussions, sample did not experience recent concussion exposure (retired athletes)	Retired athletes RPs reported more lifetime concussions than OS, *p* < 0.001, and a higher incidence of mild depression (PHQ-9 > 9) (9.55% vs 6.72%), *p* = 0.04. PHQ-9 score increased with the number of repeated concussions, regardless of the type of sport, *p* = 0.026. Daily alcohol consumption was reported more frequently by OS than RPs (31 vs 20%), but more RPs reported heavy drinking (5 glasses a day) than OS (88 vs 71%)
Didehbani et al. [19]	*n* = 29 (50%)^a^; mean concussions for players = 3.97	Depression (BDI-II)	Self-reported lifetime concussions, sample did not experience recent concussion exposure (retired athletes)	Retired athletes had higher total BDI-II scores (mean = 8.80, SD = 8.33) than control group (mean = 2.83, SD = 3.95), *p* < 0.001. In retired athletes lifetime concussions and total scores on the BDI-II were significantly correlated (*r* = 0.43, *p* = 0.02) and remained significant when cardiovascular risk factors, headaches, and arthritis were entered as covariates
Guskiewicz et al. [20]	*n* = 1513 (60.7%)^a^; mean concussions = NR	Depression (self-reported history of diagnosed depression)	Self-reported lifetime concussions, sample did not experience recent concussion exposure (retired athletes)	Retired athletes reported a retrospective self-reported lifetime depression diagnosis rate of 11.1% (of the 2434 respondents). There was an association between recurrent concussion and diagnosis of depression, *p* < 0.005, with a significant test for linear trend suggesting that the prevalence increases in a linear fashion with increasing concussion history. Relative to retired athletes with no concussion history, retired athletes reporting a history of ≥ 3 previous concussions were three times more likely (prevalence ratio of 3.06) to be diagnosed with depression, and those with a history of 1 or 2 previous concussions were 1.5 times more likely (prevalence ratio of 1.48; 95% CI 1.08–2.02) to have been diagnosed with depression
Hart et al. [21]	*n* = 34 (94.1%)^a^; mean concussions = 4.0	Depression (clinician diagnosis, BDI-II)	Self-reported lifetime concussions, sample did not experience recent concussion exposure (retired athletes)	A quarter (*n* = 8, 24%) of the retired athletes were diagnosed with major depression, 6 of whom had not been previously diagnosed or treated. The prevalence of depression amongst the retired players (24%) was higher than expected for this age group (approximately 15%)
Hutchison et al. [22]	*n* = 20 (37.7%); mean concussions = 0.90	Tension, depression, anger, vigour, fatigue, confusion, and self-esteem (POMS)	Within 4 days, 1 and 2 weeks post-concussion, included a baseline assessment	Athletes experienced short-term emotional reactions after concussion that were different from that of musculoskeletal injury. Factorial ANOVAs indicated that concussion produced an emotional profile characterised by significantly elevated fatigue and decreased vigour, and short-term mood disturbance. In contrast, musculoskeletal injury was associated with anger
Kerr et al. [23]	*n* = 307 (38.8%)^a^; mean concussions = NR	Mental health composite (VR-12)	Self-reported lifetime concussions, sample did not experience recent concussion exposure (retired athletes)	There was a non-significant trend level association (*p* = 0.06) reported between number of concussions reported by retired athletes and their mental health composite scores. Those without a concussion history reported marginally higher (i.e. better) mental health scores (mean = 53.4, SD = 5.5), relative to those with 1–2 concussions (mean = 50.7, SD = 10.1), or ≥ 3 concussions (mean = 50.4, SD = 10.5)
Kerr et al. [24]^b^	*n* = 307 (38.8%)^a^; mean concussions = NR	Depression (PHQ-9), impulsivity (BIS-15), aggression (BPAQ-SF)	Self-reported lifetime concussions, sample did not experience recent concussion exposure (retired athletes)	In retired athletes reporting ≥ 3 concussions, the prevalence of moderate-to-severe depression was 2.4 times that of former collegiate athletes reporting zero concussions (95% CI 1.0–5.7; controlling for alcohol dependence, family history of depression). Those reporting ≥ 2 or ≥ 3 concussions had significantly higher mean impulsivity scores compared to those reporting no concussions. Similarly, those reporting ≥ 3 concussions had significantly higher mean score for aggression, compared to those reporting no concussions
Kerr et al. [25]	*n* = 679 (65.0%)^a^; mean concussions = NR	Depression (self-reported history of diagnosed depression), wellbeing (SF-36)	Self-reported lifetime concussions, sample did not experience recent concussion exposure (retired athletes), mental health assessed at baseline and follow-up (9 years post-baseline)	Retired athletes self-reporting concussions were at greater risk of experiencing depressive episodes during the 9-year follow-up relative to retired athletes self-reporting no concussions. A total of *n* = 106 (10.2%) reported receiving a diagnosis of depression between the baseline and follow-up period. The 9-year risk of a depression diagnosis increased with number of self-reported concussions, ranging from 3.0% in the “no concussions” group to 26.8% in the “10+” group (*p* < 0.001). A strong dose-response relationship was observed even after controlling for confounders
Kontos et al. [26]	*n* = 75(100%); mean concussions = 1.08 (prior to current exposure)	Depression (BDI-II)	Within 2, 7, 14 days post-concussion, included a baseline assessment	Athletes post-concussion exhibited significantly higher levels of depression as assessed on the BDI between baseline (mean = 1.68, SD = 2.11), and at 2 days (mean = 4.52, SD = 4.46), 7 days (mean = 4.21, SD = 5.61), and 14 days (mean = 5.21, SD = 7.00), *p* < 0.05
Mainwaring et al. [27]	*n* = 16 (31.4%); mean concussions = 1.4	Tension, depression, anger, vigour, fatigue, confusion, and self-esteem (POMS)	Within 4, 7, 14 days post-concussion, included a baseline assessment	Pre-injury POMS performance in athletes was not a risk factor for concussion. There was a significant acute spike for depression, confusion, and total mood disturbance at 4 days post-concussion. These increases were transient and appeared to resolve by 14 days
Mainwaring et al. [28]^c^	*n* = 16 (5.1%); mean concussions = NR	Tension, depression, anger, vigour, fatigue, confusion, and self-esteem (POMS)	Within 4, 7, 14 days post-concussion, included a baseline assessment	Athletes with concussion and ACL injury reported significant increases in depression scores post-injury compared with uninjured controls. Athletes with ACL injury reported higher levels of depression for a longer duration than athletes with concussion
Meehan et al. [29]	*n* = 836 (22.6%)^a^; mean concussions = NR	Anxiety, depression, emotional and behavioural dyscontrol, positive affect, sleep disturbance (Neuro-QoL), alcohol use (PROMIS)	Self-reported lifetime concussions, sample did not experience recent concussion exposure (retired athletes)	Respondents with a history of concussion self-reported worse health on several measures, including positive affect, measures of anxiety, depression, negative consequences of alcohol use, sleep disturbance, emotional and behavioural dyscontrol, and fatigue (*p* values < 0.001)
Meier et al. [30]	*n* = 17(38.6%); mean concussions = 1.0 (prior to current exposure)	Anxiety (HAM-A), depression (HAM-D)	Within 3 days (T1: *n* = 17), 13 days (T2: *n* = 15), 44 days (T3: *n* = 13) post-concussion, included a baseline assessment	Compared to healthy controls, athletes with concussion showed partial recovery of clinically assessed mood symptoms by T2 and T3 relative to T1 post-concussion; however, mood symptoms remained elevated throughout subacute assessment phase. Anxiety was significantly higher for the post-concussion group relative to healthy athletes at T1 and T2 (*p* values < 0.001) and trending higher at T3 (*p* < 0.10). Depression was significantly higher for the post-concussion group relative to healthy athletes at T1, T2 (*p* values < 0.001) and T3 (*p* = 0.01). There was no significant main effect or interaction of prior concussions on recovery of anxiety or depression (*p* values > 0.10)
Meier et al. [31]	*n* = 40 (100%); mean concussions = 0.93 (prior to current exposure)	Anxiety (HAM-A), depression (HAM-D)	Within 7 days (1.92 days, SD 1.04) post-injury	Concussed athletes significantly underreported post-concussive symptoms to their athletic trainers according to the ImPACT Post-Concussion Scale. Standardised measures of depression and anxiety (*p* values < 0.001) were higher when reported in a confidential setting
Meier et al. [32]	*n* = 44 (45.7%); mean concussions = 0.75 (prior to current exposure)	Anxiety (HAM-A), depression (HAM-D)	Within 1 day (T1: 1.74, SD 0.93; *n* = 34); 7 days (T2: 8.44, SD 2.15; *n* = 34); 30 days (T3: 32.47, SD 4.68; *n* = 30) post-concussion	Concussed athletes showed improvement in mood symptoms at each time point, but had significantly higher mood scores than healthy athletes at every time point; concussed athletes had higher depression scores relative to healthy athletes at T1 and T2 (*p* values < 0.001) and T3 (*p* = 0.003). Concussed athletes had higher anxiety scores relative to healthy athletes at T1, T2 (*p* values < 0.001) and T3 (*p* = 0.033)
Montenigro et al. [33]	*n* = NR^a^; mean concussions = NR; median concussions = 20	Depression (CES-D), apathy (AES)	Self-reported lifetime concussions, sample did not experience recent concussion exposure (retired athletes)	The cumulative head impact index predicted later-life clinical outcomes, outperforming other individual metrics such as concussion history, age at first exposure to American Football, and total duration of play. A dose-response relationship between estimated cumulative head impact exposure all and later-life risk for neurobehavioral impairment was observed. Risk of developing behavioural dysregulation, depression, and apathy nearly doubled with 2800 additional impacts above the threshold
Poltavski and Biberdorf [34]	*n* = 17 (40%)^a^; mean concussions = NR	ADHD (ASRS)	Self-reported lifetime concussions, currently competing athletes, pre-season assessment	Athletes with a self-reported history of concussion scored significantly higher for ADHD checklist (mean = 10.24, SD = 2.77) relative to athletes without a history of concussion (mean= 7.88, SD = 4.01) (*p* = 0.04)
Pryor et al. [35]	*n* = 27 (100%)^a^; mean concussions = NR	Depression (CES-D)	Self-reported lifetime concussions, currently competing athletes, pre-season assessment	Individuals with a CES-D score of ≥ 16 sustained a significantly greater number of lifetime concussions (3.8 vs 1.6) (*p* < 0.001). Significantly higher CES-D scores were observed in players who had sustained ≥ 3 concussions (24.0 vs 15.6) than those with ≤ 2 (*p* = 0.03)
Putukian et al. [36]	*n* = 32 (12.2%); mean concussions = NR	Anxiety (GAD-7), depression (PHQ-9)	Baseline (T1) and post-concussion (T2; mean = 283.3, SD = 259.6 days)	There was no significant interaction (*p* values < 0.1) between time of assessment (T1, T2) and group (concussed, control) for either anxiety or depression. Main effects were not reported
Roiger et al. [37]	*n* = 7 (33.3%); mean concussions = NR	Depression (CES-D)	1 week, 1 and 3 months post-concussion, included a baseline assessment	Concussed athletes had higher depression symptoms 1 week post-concussion (mean = 11.0, SD = 5.3) compared to baseline (mean = 6.7, SD = 3.9), *p* = 0.02. There were no significant differences between baseline depression and depression at 1 or 3 months. There were no differences in depression between the concussed and injured groups at any time point
Singh et al. [38]	*n* = 18 (50%); mean concussions = 1.11 (prior to current exposure)	Anxiety (HAM-A), depression (HAM-D)	3 days, 1 week, 1 month post-concussion	Concussed players reported significantly higher depression and anxiety at 3 days (*p* values < 0.001), and at 1 week (*p* values < 0.001), post-concussion relative to healthy athletes. At 1-month, depression remained higher in the post-concussion group (*p* = 0.041), though there was no difference between the groups at this time for anxiety
Strain et al. [39]	*n* = NR^a^; mean concussions = 3.43 (asymptomatic group); mean concussions = 5.6 (symptomatic group)	Depression (BDI-II)	Self-reported lifetime concussions, sample did not experience recent concussion exposure (retired athletes)	Of the 26 retired athlete participants, 5 (19.2%) were identified as currently symptomatic for depression (scoring > 18 on the BDI-II). Those in the symptomatic group reported on average 5.6 (SD = 3.29) lifetime concussions, relative to 3.43 (SD = 2.87) for those asymptomatic, although this difference was not statistically significant
Vargas et al. [40]	*n* = 84 (65.6%); mean concussions = 0.92 (prior to current exposure)	Depression (BDI-FS)	2 days (*n* = 36), 5 days (*n* = 60), 1–7 weeks (*n* = 19) post-concussion, included a baseline assessment, controls re-assessed mean = 6.8 weeks post-baseline	For those in the post-concussion group, a total of 9 of 84 athletes (11%) at baseline and 19 of 84 athletes (23%) post-concussion scored above the cut-off (BDI-FS > 3) (*p* = 0.02). The difference between T1 and T2 for the controls was not significant (*n* = 3; 7% vs *n* = 4; 10%), *p* > 0.9. Significant baseline covariates of post-concussion depression were baseline depression (*p* = 0.03) and age first played sport (*p* = 0.005)
Yang et al. [41]	*n* = 67 (100%); mean concussions = NR	Anxiety (STAI), depression (CES-D)	Within 1 week, and depending on injury duration also 1, 3, 6, 9, 12 months post-concussion, included a baseline assessment	Concussed athletes who had symptoms of depression at baseline (pre-injury) were 4.59 times more likely (95% CI 1.25–16.89) to experience depression symptoms and 3.40 times more likely (95% CI 1.11–10.49) to experience state anxiety following the concussion, compared to concussed athletes who had no symptoms of depression at baseline. Concussed athletes with baseline (pre-injury) trait anxiety did not have increased post-concussion depression and state anxiety symptoms. Post-concussion symptoms of depression significantly co-occurred with post-concussion state anxiety (OR = 8.35, 95% CI 2.09–33.34)

*ACL* anterior cruciate ligament, *ADHD* attention deficit hyperactivity disorder, *AES* Apathy Evaluation Scale, *ANOVA* analysis of variance, *ASRS* Adult ADHD Self-Report Scale, *BDI* Beck Depression Inventory, *BDI-FS* Beck Depression Inventory—Fast Screen, *BDI-II* Beck Depression Inventory 2, *BIS-15* Barrett Impulsiveness Scale, *BPAQ-SF* Buss-Perry Aggression Questionnaire—Short Form, *CES-D* Center for Epidemiologic Studies Depression Scale, *CI* confidence interval, *GAD-7* Generalized Anxiety Disorder-7 Item Scale, *HAM-A* Hamilton Anxiety Rating Scale, *HAM-D* Hamilton Depression Rating Scale, *ImPACT* Immediate Post-Concussion Assessment and Cognitive Test, *MMA* mixed martial arts, *Neuro-QoL* Quality of Life in Neurological Disorders, *NR* not reported, *OR* odds ratio, *OS* other sports, *PHQ-9* Patient Health Questionnaire, *POMS* Profile of Mood States, *PROMIS* Patient-Reported Outcomes Measurement Information System, *RP* rugby player, *SD* standard deviation, *SF-36* Short Form 36 Measurement Model for Functional Assessment of Health and Well-Being, *STAI* State-Trait Anxiety Inventory, *T1* time 1, *T2* time 2, *T3* time 3, *VR-12* Veterans RAND 12-Item Health Survey

^a^Retrospective report of concussion (i.e. > 4 weeks elapsed between concussion exposure and mental health assessment) or unable to determine time elapsed between exposure and assessment

^b^Same sample as Kerr et al. [23]

^c^Same sample as Mainwaring et al. [27]

With the exception of one study conducted in France, all other studies were from North America (USA 85.2%, *n* = 23 studies; Canada 11.1%, *n* = 3). Almost half the included studies (44.4%, *n* = 12) reported on data from male-only samples. A variety of study designs were utilised, including cross-sectional, with and without a comparison group (55.6%, *n* = 15), prospective cohort (25.9%, *n* = 7), mixed longitudinal and cross-sectional (7.4%, *n* = 2), prospective case-control (7.4%, *n* = 2), longitudinal (7.4%, *n* = 2) and cohort studies (3.7%, *n* = 1). There were no randomised controlled trials within the included studies. The included studies examined athletes from a broad range of individual sports (e.g. swimming, diving, wrestling, boxing) and team-based sports (e.g. American Football, hockey, soccer, rugby), with many studies including elite athletes from a range of sports. The majority of studies were conducted with North American college athletes (66.7%, *n* *=* 18). The sample size of the included studies ranged from 21 to 3656. The follow-up period for the prospective and longitudinal studies ranged markedly from 2 days to 29 years.

### Quality Appraisal

All 27 studies included in the review were assessed for methodological rigour using the Quality Assessment Tool for Observational Cohort and Cross Sectional Studies published by the US National Institutes of Health (2014) [42]. The tool comprises 14 criteria (see Electronic Supplementary Material Table S1). An additional criterion was added to determine whether the study included a statement regarding the sample being free of the outcome of interest (in this instance a mental health outcome) at exposure. A final score was calculated for each study as the percentage of criteria met. Percentages were categorised according to previously published guidelines [43, 44]. Each study was rated as excellent (75–100%; very low risk of bias), good (50–74%; most methodological criteria met, low risk of bias), fair (25–50%; some criteria met, possible risk of bias), or poor (0–25%; few criteria met, high risk of bias).

The quality of the included studies varied, with final scores ranging between 28% and 77%. The mean quality rating was 53.8%. In total, one study was rated as excellent (3.7%), 15 studies were rated as good (55.6%), 11 studies were rated as fair (40.7%), and no studies were rated as poor. All included studies reported clear research objectives and most reported clearly defined population (88.9%, *n* = 24) and mental health outcomes (96.3%, *n* = 26). However, the majority of studies included in the analysis did not measure or report on concussion history prior to the onset of any mental health outcomes, such as previous concussions that may have been experienced during childhood or adolescence (92.6%, *n* = 25), and only one fifth of studies (*n* = 6) reported that the sample was assessed as free of the mental health outcome at the time of exposure (concussion). Inference of a temporal association was not possible in half of the studies (51.9%, *n* = 14), and concussion was assessed at more than one time point in only one of the included studies (3.7%).

### Main Findings

Study outcome variables and outcomes measures are summarised in Table 3. Findings are discussed below relative to mental health outcome and athlete population. Outcomes are reported separately for depression, anxiety, multi-domain symptom assessment, ADHD, and finally, impulsivity, apathy and aggression.

#### Depression

As indicated in Sect. 3.2, depression symptoms were the most frequently assessed mental health outcome of the included studies. The findings for depression symptoms are organised below according to (a) studies reporting on retired athlete populations, followed by (b) studies reporting on currently competing athletes.

Of the 19 studies reporting depression symptoms as an outcome, almost half (47.4%, *n* = 9) involved samples of retired athletes. The majority of these (*n* = 6 studies) involved retired American National Football League (NFL) players. In these studies, the reported rates of depression symptoms varied markedly relative to concussion exposure. For example, in two small studies that used self-report measures, rates of depression meeting clinical cut-off were 33% (total *n* = 45) [16] and 19.2% (total *n* = 26) [39], whereas in larger samples that assessed rates of previous clinician diagnosis of depression, the rates were 11.1% (total *n* = 2434) [20] and 10.2% (total *n* = 1044) [25] (community 12-month prevalence of diagnosed depression approximates 7.5% [45]). Of the included studies, Hart et al. [21] was the only one to use formal mental health diagnosis (via a clinical interview), with 24% (*n* = 8) meeting clinical diagnostic criteria (total *n* = 34). Another smaller study reported a dose-response relationship between cumulative head impact and later-life risk for depression symptoms (total *n* = 76) [33], with this response also observed in Didehbani et al.’s study [19], which reported a significant moderate correlation (*r* = 0.43) between number of concussions and self-reported depression (total *n* = 59).

This dose response between number of concussions and depression symptoms was also reported in the remaining two studies with retired (non-NFL) athletes. Self-reported depression severity increased with the number of reported concussions and was higher in those who had played rugby relative to other sports (total *n* = 377) [18]. In a large, mixed sports sample (involving basketball, baseball, wrestling), those reporting three or more concussions were 2.4 times more likely than those reporting no concussion to experience moderate-severe depression (total *n* = 797) [24].

The remaining ten studies were conducted in athletes in current competition. Of these, over half (60%; *n* = 6) included a baseline (i.e. pre-concussion) assessment, enabling a comparison of relative pre-morbid symptoms prior to recent concussion exposure, and four included a non-concussed control comparison. One study (*n* = 44) found that relative to healthy controls, concussed American college footballers exhibited partial, but not full, recovery of clinically assessed mood symptoms by 13 and 44 days [30]. Another study (total *n* = 263) in a mixed sports sample of athletes found no interaction between group (i.e. concussion, healthy control) and time of assessment (baseline, follow-up); however, this study included a long-term follow-up period (mean 283 days) between concussion exposure and depression symptoms [36]. The third study (total *n* = 84), also in a mixed sports sample of athletes, found that compared to baseline (11%), concussed athletes were significantly more likely to experience threshold symptoms of depression at follow-up (23%), while depression rates did not differ from baseline to follow-up for the control group [40]. Another study in a mixed sports sample (total *n* = 67) found that concussed athletes who reported symptoms of depression at baseline (e.g. pre-injury) were 4.59 times more likely to experience post-concussion depression symptoms than concussed athletes not reporting depression symptoms at baseline [41]. With the exception of the study employing the longer-term follow-up [36], relative to healthy controls, there is some evidence for elevated symptoms of depression post-concussion, although this may be confined to the short–medium term only, and may be influence by pre-morbid depression. The two small studies (both *n* = 21) reporting depression symptoms without a control comparison (both in mixed sports samples) indicated findings consistent with the controlled studies above. In one study, concussed athletes reported significantly elevated depression scores relative to their baseline, at 2, 7 and 14 days post-concussion [26], with the other study reporting a significant difference between baseline and assessment at week 1 post-concussion, but not at 1 or 3 months [37].

The remaining four studies reporting post-concussion depression symptoms in currently competing athletes did not include a baseline assessment, though two included a comparison with healthy controls. These two controlled studies reported similar results to the studies described above, where concussed athletes (mixed sports sample) showed improvement in mood symptoms at each time point, but had significantly higher (i.e. worse) mood scores than healthy athletes at 1 week and 1 month post-concussion (total *n* = 94) [32]; this mirrored the results for the small sample of American college footballers at 3 days, 1 week, and 1 month post-concussion (total *n* = 36) [38]. The remaining two studies reporting depression symptoms in currently competing athletes found that in a mixed sample of professional (NFL) and semi-professional American footballers, those with a history of three or more concussions reported significantly higher depression symptoms than those with two or fewer concussions (total *n* = 27) [35]. A separate sample of mixed sports athletes demonstrated a tendency for under-reporting of post-concussive mood symptoms when symptoms reported to coaching staff were compared to those reported on a confidential standardised depression scale (total *n* = 40) [31].

#### Anxiety

There was also variability in the outcomes for anxiety symptoms. Seven studies reported anxiety symptoms as an outcome, all of which were conducted in currently competing athletes. Of the three studies that included a baseline assessment, two included a healthy control comparison. One of these studies (*n* = 44) found significantly higher anxiety symptoms (assessed by structured clinical interview) in post-concussed American college footballers at 13 days (but not at 44 days) relative to healthy controls [30]. Consistent with this, the other study with a mixed sports sample (*n* = 263) with a longer-term follow-up (283 days) reported no significant interaction for anxiety symptoms between time of assessment and concussed versus healthy controls [36]. The third of these studies reported that in a mixed sports sample (*n* = 67), those with depression at baseline were 3.4 times more likely to experience state anxiety symptoms post-concussion than athletes without depression at baseline [41]. Two studies included healthy controls, but not a baseline assessment. These studies reported that relative to healthy controls, concussed American college footballers reported significantly higher anxiety symptoms at 3 days and at 1 week, though not at 1 month (*n* = 36) [38], and that in a mixed sports sample, concussed athletes reported higher anxiety scores at 1 day, 1 week and 1 month (*n* = 94) [32]. A further anxiety study in a mixed sports sample found equivalent rates of anxiety symptoms in athletes with recent concussion and orthopaedic injury and little difference between the groups in sources of social support sought during injury (*n* = 126) [17]. The final study (in a mixed sports sample) found that post-concussion, athletes under-reported anxiety symptoms to coaching staff relative to self-reported anxiety (using a standardised rating scale) (*n* = 40) [31].

#### Multi-domain Measures

Five studies reported on broad multiple domain outcomes for mental health. Four studies used the POMS, of which two were conducted in current athletes, and included a baseline assessment prior to recent concussion exposure. One of these studies also included a healthy control comparison, and reported that in a mixed sports sample (*n* = 51), athletes with either concussion or anterior cruciate ligament (ACL) injury reported significant increases in depression symptom scores post-injury compared with un-injured controls, though athletes with ACL injury reported higher levels of depression for a longer duration than athletes with concussion [27]. In a larger sample of mixed sports athletes (*n* = 369), a significant acute increase in depression symptoms, confusion, and total mood disturbance was seen at 4 days post-concussion, although these increases were transient and resolved by 14 days [28]. In the third study of mixed sports athletes (*n* = 53), concussion was associated with a short-term (2-week) profile characterised by elevated fatigue and decreased vigour, and short-term mood disturbance, whereas musculoskeletal injury was associated with anger [22]. The remaining two studies were cross-sectional, reporting multi-domain outcomes in samples of retired athletes from mixed sports. Respondents with a history of concussion self-reported worse outcomes on several measures, including positive affect, negative consequences of alcohol use, sleep disturbance, emotional and behavioural dyscontrol and fatigue (*n* = 3656) [29]. Similarly, those without a concussion history reported better composite mental health scores relative to those with either one to two concussions or three or more concussions (*n* = 797) [23].

#### Attention Deficit Hyperactivity Disorder

One study reporting on ADHD was identified. This study found that athletes with a self-reported history of concussion scored significantly higher on an ADHD checklist relative to athletes without a history of concussion (total *n* = 42) [34].

#### Impulsivity, Aggression, Apathy

The remaining mental health outcomes that were assessed in the included studies were impulsivity (*n* = 2), aggression (*n* = 1) and apathy (*n* = 1). In the studies reporting impulsivity, currently competing boxers (mean concussions = 0.4) and mixed martial arts athletes (mean concussions = 0.6) reported lower impulsiveness and self-control scores relative to healthy male controls (*n* = 131) [15], whereas a sample of retired athletes from mixed sports with two or more reported concussions had significantly higher mean impulsivity scores compared to those reporting no concussions (*n* = 797) [23]. This same study also found that those reporting three or more concussions had a significantly higher mean score for aggression, compared to those reporting no concussions [23]. The study reporting apathy was conducted in retired American college footballers and found that risk of higher apathy scores increased with cumulative head impacts (total *n* = 76) [33].

#### Control Groups Used

In total, ten of the included studies drew comparisons with a participant control group. In one study, the control condition included orthopaedic injury, and was matched according to sex, sport, and time lost due to injury [17]. In the remaining nine studies, the control condition was broadly characterised as ‘healthy’. Of these nine studies, three reported comparison data from a physically active uninjured group of similar age peers [22, 28, 36]. The remaining six reported some level of specific matching controls (i.e. age, sex, education) [15, 18, 19, 30, 32, 38]. One of these studies also matched on estimated IQ [19]. Of the seven longitudinal studies using a control group, four reported baseline comparison only (i.e. no control group longitudinal data reported) [15, 30, 32, 38]. For the remaining three studies, there was no observable change in mood symptoms over time for uninjured athletes [22, 28, 36].

## Discussion

This review is the most comprehensive to date to appraise the extant literature regarding concussion and mental health outcomes amongst elite athletes. The findings extend those of Finkbeiner and colleagues [11] and Manley and colleagues [12] by involving a more comprehensive screening process (14,960 abstracts) and focussing on sport-related concussion in adult *elite* athletes, both competing and retired. Consistent with the previous reviews, we found evidence of acute/subacute associations between concussion and depression symptoms. There was some evidence of longer-term effects for elevated depression symptoms in retired athletes [16, 21, 39], though this effect was less pronounced for studies that reported on larger samples [20, 25]. Emerging evidence of a dose-response relationship between concussion exposure and depression symptoms is worthy of further study [18, 19, 24]. For other mental health or symptom domains, the evidence was less consistent, or there were insufficient studies available. The robustness of conclusions that can be drawn from this review must be tempered, however, by the methodological limitations of studies to date. These include a paucity of representative studies using a prospective, longitudinal design that measures both the dependent and independent variables at baseline (mental health and concussion history) and involves a non-concussed comparison group, along with other relevant baseline predictor variables (particularly those known to be associated with poor mental health, such as inadequate social support, coping style, adverse life events). With these limitations in mind, we evaluate the findings relevant to symptom domains and offer recommendations for research in order to improve rigour, enabling firmer conclusions to be drawn in the future, especially in relation to the temporal relationship between concussion and mental health outcomes.

### Depression

Depression symptoms were the most frequently considered and reported mental health outcome in the concussion research. Almost half of these studies (typically involving the largest samples) have involved retired athletes, and rely upon retrospective recall of concussion. Relying on retrospective recall (i.e. self-reported history) of concussion exposure is problematic as it may be prone to memory bias and inaccurate self-diagnosis. Further, often prolonged intervals elapsed between the concussion exposure and reporting of depression symptoms, which may be mediated by other factors that are not reliably controlled for, such as adverse life events, substance use and social support. These cross-sectional studies generally (but not consistently) show a link between lifetime concussion and elevated self-reported symptoms of depression [16, 18, 19, 21, 24, 33, 39], including a dose-response in studies that have assessed the number of concussions. The relationship between concussion and depression symptoms is also observed in the smaller controlled studies, where six of eight studies found a persistent (i.e. 4-week) effect of elevated depression in post-concussion athletes relative to healthy (non-concussed) controls.

### Anxiety

Relative to depression symptoms, the studies reporting on symptoms of anxiety showed less consistent findings. The two controlled studies that involved a baseline assessment failed to find a persistent (i.e. > 1-month) effect for anxiety symptoms post-concussion [30, 36], and a similar finding was noted in a controlled study without a baseline assessment [38], although a fourth controlled study without a baseline assessment found concussed athletes did report significantly higher anxiety symptoms at 4 weeks [32]. With relatively few studies, conclusions related to the link between concussion exposure and anxiety symptoms are premature. Further studies are required that also consider the *type* of anxiety that may be associated with concussion (e.g. panic attacks/symptoms, generalised anxiety, or health anxiety, the latter including fears of not regaining pre-concussion functions, or susceptibility to further injury or impairment).

### Other Outcomes

Studies employing multi-domain measures were also inconsistent, in part because of the variation in measures used. In some studies, associations were observed across multiple domains following concussion, such as confusion and mood disturbance [28], fatigue and vigour [22], and alcohol use and sleep disturbance [29]. The two studies reporting impulsivity were mixed [15, 23], and little can be concluded from the studies reporting on ADHD [34], aggression [23], and apathy [33]. To date, there are no published studies on other mental health outcomes of concussion, such as problematic alcohol or substance use, psychotic-like symptoms (e.g. paranoid beliefs, visual or auditory hallucinations), or personality disturbance.

### Implications

The findings of this review complement current guidelines for the management of sport-related concussion that recommend that any prolonged concussion recovery must include monitoring of mental health symptoms, in particular depression [46, 47]. In contextualising our results, it is important to reflect on how sport-related concussion may induce mental health symptoms—or vice versa—and at present, the mechanistic link between these constructs is poorly understood [48]. Outside of boxing, where a link between repeated head impact and chronic traumatic encephalopathy has been established [49], a direct association between exposure to sport-related concussion and longer-term neuro-affective effects (i.e. depression, suicidality) is not established [50]. This is also the case for structural injuries, changes to neural signalling, and biochemical immunoexcitotoxic mechanisms, all of which have been proposed as possible mechanisms of actions [51–53]. The growing literature on sub-concussive blows in combat veterans with military service history may be relevant to advancing understanding in the sports medicine field [48]. There also needs to be greater consideration of the extent to which mental health difficulties may increase *susceptibility to concussion* in elite athletes (e.g. via increased distraction or preoccupation with emotional distress), a topic which has yet to be adequately investigated in high-quality research. Further, previous literature has emphasised the lack of specificity between post-concussion symptoms and symptoms of depression and anxiety [54, 55]. For example, the SCAT-5 includes assessment of difficulty concentrating, fatigue, sleep disturbance, sadness, irritability and anxiety [10], which overlap considerably with symptoms assessed in measures of depression and anxiety used in the included studies. Several of the included longitudinal studies attempted to deal with this issue statistically [26, 40, 41]. However, lacking in the current literature are well-designed longitudinal studies that control for baseline mental health (i.e. symptoms of depression and anxiety) prior to concussion exposure and include adequate follow-up assessment (ideally over months and years) for subsequent symptoms of depression and anxiety.

Recently, Asken and colleagues [51] proposed a biopsychosocial model for negative outcomes in sport-related concussion for athletes likely to experience collision sport exposure. This model includes pathways by which salient neurobiological (i.e. genetic factors, normal aging, anaesthesia exposure) and psychosocial factors (i.e. social withdrawal, transition and adjustment, financial status, substance use) may mediate and moderate mental health outcomes. Given that mental health indicators, such as depression, are heterogeneous and multifactorial in nature, a biopsychosocial approach has face validity. It is also possible that key variables such as retirement [56] and injury [57] may mediate the potential relationship between concussion exposure and mental health outcomes, such as depression. A recent analysis of suicide in NFL players found that major life stressors, mental health problems, substance abuse and medical problems pre-dated their deaths [50]. Assessing risk and vulnerability factors separate to concussion exposure is critical. Recent research in young athletes has shown that pre-morbid mental health status (i.e. pre-morbid mood disorder or other psychiatric history including ADHD and impulsivity), previous concussion history, and indeed life stressors predict the number of concussions experienced [58, 59] and the development of post-concussive syndrome lasting > 3 months [60], demonstrating that factors outside of exposure per se should be carefully considered. Furthermore, as susceptibility to concussion may have a genetic component [61, 62], assessment of genetic factors or biomarkers is also indicated in future research.

### Recommendations for Research

The understanding and appreciation of the relationship between sport-related concussion and mental health are rapidly emerging, as evidenced by the explosion in research in this area since 2014 (consisting of 70% of the studies included in this review). It is likely that the next decade will see substantial gains in the knowledge base related to the sequelae of sport-related concussion and its management. However, the methodologies applied in most of the extant research are poorly suited to answering the key clinical questions of (a) is sport-related concussion associated with immediate and longer-term mental health difficulties; (b) if yes, how, and what is the temporal relationship between these factors; (c) what factors mediate or moderate this relationship; and (d) what is the best approach to prevent or manage these outcomes? There is a risk that in the absence of methodically rigorous research, ‘myths’ regarding the link between concussion and long-term health effects, including mental health, may precede scientific fact [63]. As a minimum, studies are needed that assess whether participants are free of the outcome of interest (i.e. mental health symptoms) prior to concussion exposure, and that provide an accurate (i.e. clinician or medical-record verified) concussion history prior to most recent exposure. Furthermore, no research has included a truly representative sample of a playing/competitive cohort (e.g. > 70% participation rate for particular competition/league), which is necessary to counter sample or reporting bias. Greater attention is needed to ensure diversity of study participants by ethnicity, sex and sport type.

While existing data appear to support a link between concussion exposure and elevated short-term depression symptoms, research designs based on retrospective recall in retired samples significantly limit the conclusions that can be drawn. Retrospective studies are vulnerable to memory bias, whereby athletes with depression may be more likely to inflate recall of concussive episodes or misperceive pre-morbid functioning as better than the average person [64] along with the salience of intervening factors in the athlete’s life subsequent to concussion exposure. Emphasis should instead be placed on well-designed prospective studies that can conclusively evaluate the existence of post-concussive mental health effects, and which are sensitive to symptom onset relative to concussion exposure. Exposure to non-sport–related concussion (i.e. accidents, injuries or vehicle accidents), which are the most common cause of concussions [65, 66], should also ideally be considered in study designs.

Only one of the included studies used clinician diagnosis when assessing mental health outcome [21], and only four of the included studies used a structured clinical interview when assessing outcome [30–32, 38]. Structured clinical interviews are considered the gold standard for the assessment of mental illness, and their relative lack of use to date is concerning. It is recommended that the next generation of studies include structured clinical interviews and evaluate concordance between self-report and interviewer-administered assessments post-concussion.

Finally, the majority of studies included in this review evaluated contact sports (typically American Football), with most recruiting male-only samples. This leaves gaps in our understanding of the ways in which concussion may present in other sports where head impacts occur (i.e. soccer) and in female athletes (particularly as the rates of depression and anxiety in the community are elevated amongst females compared to males). As sex may influence post-concussive symptoms [7, 67], studies must include representative female samples and explore potential sex effects. In particular, equestrian sports may be a fertile avenue for concussion research given the high prevalence of concussion in this population [68] and the relative balance of males and females who participate [61].

### Study Limitations

This review considered research published only in English language, such that relevant studies conducted in non-English samples have been overlooked. We also excluded qualitative studies, which can be a rich source of information regarding putative mechanisms of action. Nonetheless, the broad search strategy utilised here ensures a degree of confidence that, within the inclusion criteria parameters, all salient research has been identified and appraised.

## Conclusions

Regardless of the direction of the relationship, our findings and previous research [69] indicate that amongst elite athletes, both currently competing or retired, concussion and impaired mental health, particularly depression symptoms, are associated. It is essential that procedures are developed and routinely implemented to adequately screen for and detect such difficulties, and that affected individuals/athletes are provided with appropriate interventions to restore both their physical *and* mental health. The interplay of elite sport and mental health is of growing research and clinical interest [70–75]. That the association found in elite athletes may also apply to amateur or community level sports (especially contact sports) has broader implications for how concussion is (ideally) prevented, assessed and managed across all participation levels. Advances in preventing, assessing and managing concussion-related mental health problems can only be underpinned, however, by high quality research, which is currently lacking, but urgently required.

## Electronic supplementary material

Below is the link to the electronic supplementary material.

m 1 (DOCX 19 kb)
