# Supplementary material for: Sport-Related Concussion and Mental Health Outcomes in Elite Athletes: A Systematic Review
Source: Sports Med. 2017 Nov 20;48(2):447–65. doi: 10.1007/s40279-017-0810-3 (PMC5775382; doi:10.1007/s40279-017-0810-3)
Supplement: Supplementary file 1 — m 1 (DOCX 19 kb) [file 40279_2017_810_MOESM1_ESM.docx]

**Supplementary Table:** Quality appraisal of study methodology and reporting

| Study | Research question / objective clearly stated | Pop. clearly defined | Sample free of outcome (MH) at exposure | Particip.  Rate ≥50% | Participants  Recruited from similar populations | Variance or effect estimates reported | Exposure measured prior to MH outcome | Temporal association inference possible | Different levels of exposure measured | Exposure measures clearly defined | Exposure assessed more than once | Outcome measures (MH) clearly defined | Blinded outcome assessors | Loss to follow-up ≤20% | Models adjusted for at least one confound | **N(%) of items^#^ addressed or reported** |
| --- | --- | --- | --- | --- | --- | --- | --- | --- | --- | --- | --- | --- | --- | --- | --- | --- |
| Banks et al. [15] | ✓ | **×** | NR | NR | ✓ | ✓ | **×** | **×** | **×** | **×** | **×** | ✓ | **×** | NA* | **×** | 4 (**28%**) |
| Casson et al. [16] | ✓ | ✓ | **×** | **×** | ✓ | **×** | **×** | **×** | ✓ | **×** | **×** | ✓ | NA* | NA* | **×** | 5 (**38%**) |
| Covassin et al. [17] | ✓ | ✓ | NR | NR | ✓ | ✓ | **×** | ✓ | **×** | **×** | **×** | ✓ | NR | NA* | **×** | 6 (**43%**) |
| Decq et al.[18] | ✓ | ✓ | NR | **×** | ✓ | ✓ | **×** | **×** | ✓ | **×** | **×** | ✓ | NR | NA* | **×** | 6 (**43%**) |
| Didehbani et al. [19] | ✓ | ✓ | NR | NR | CD | ✓ | **×** | **×** | ✓ | **×** | **×** | ✓ | NR | NA* | ✓ | 6 (**43%**) |
| Guskiewicz et al. [20] | ✓ | ✓ | NR | ✓ | ✓ | ✓ | **×** | **×** | ✓ | **×** | **×** | ✓ | NR | NA* | ✓ | 8 (**57%**) |
| Hart et al. [21] | ✓ | ✓ | NR | NR | ✓ | ✓ | **×** | **×** | ✓ | **×** | **×** | ✓ | NR | NA* | **×** | 6 (**43%**) |
| Hutchison et al. [22] | ✓ | ✓ | NR | ✓ | ✓ | ✓ | **×** | ✓ | **×** | ✓ | **×** | ✓ | NA* | NR | **×** | 8 (**57%**) |
| Kerr et al. [23] | ✓ | ✓ | NR | **×** | ✓ | ✓ | **×** | **×** | ✓ | **×** | **×** | ✓ | NA* | NA* | **×** | 6 (**46%**) |
| Kerr et al. [24]^a^ | ✓ | ✓ | NR | **×** | ✓ | ✓ | **×** | **×** | ✓ | **×** | **×** | ✓ | NA* | NA* | **×** | 6 (**46%**) |
| Kerr et al. [25] | ✓ | ✓ | NR | ✓ | ✓ | ✓ | **×** | **×** | ✓ | **×** | **×** | ✓ | NA* | NA* | ✓ | 8 (**62%**) |
| Kontos et al. [26] | ✓ | ✓ | **×** | NR | ✓ | ✓ | **×** | ✓ | ✓ | ✓ | **×** | ✓ | **×** | NR | **×** | 8 (**53%**) |
| Mainwaring et al., [27] | ✓ | ✓ | ✓ | NR | ✓ | ✓ | **×** | ✓ | ✓ | ✓ | **×** | ✓ | NA* | NA* | **×** | 9 (**69%**) |
| Mainwaring et al., [28]^b^ | ✓ | ✓ | ✓ | NR | ✓ | ✓ | ✓ | ✓ | ✓ | ✓ | **×** | ✓ | NA* | NA* | **×** | 10 (**77%**) |
| Meehan et al. [29] | ✓ | ✓ | NR | NR | ✓ | ✓ | **×** | **×** | ✓ | **×** | **×** | ✓ | NA* | NA* | ✓ | 7 (**54%**) |
| Meier et al. [30] | ✓ | ✓ | NR | NR | ✓ | ✓ | **×** | ✓ | ✓ | ✓ | **×** | ✓ | NA* | NA* | **×** | 8 (**62%**) |
| Meier et al. [31] | ✓ | ✓ | NR | NR | ✓ | ✓ | **×** | ✓ | ✓ | ✓ | **×** | ✓ | NA* | NA* | **×** | 8 (**62%**) |
| Meier et al. [32] | ✓ | ✓ | ✓ | **×** | ✓ | ✓ | **×** | ✓ | ✓ | ✓ | **×** | **×** | NA* | **×** | ✓ | 9 (**64%**) |
| Montenigro et al. [33] | ✓ | ✓ | NR | NA* | ✓ | ✓ | **×** | **×** | ✓ | ✓ | **×** | ✓ | NA* | NA* | ✓ | 8 (**67%**) |
| Poltavski and Beberdorf [34] | ✓ | ✓ | NR | NR | ✓ | ✓ | **×** | **×** | ✓ | NR | NR | ✓ | **×** | NA* | **×** | 5 (**36%**) |
| Pryor et al. [35] | ✓ | ✓ | NR | NR | ✓ | ✓ | **×** | **×** | ✓ | **×** | **×** | ✓ | NA* | NA* | **×** | 6 (**46%**) |
| Putukian et al. [36] | ✓ | ✓ | NR | NR | ✓ | ✓ | **×** | ✓ | ✓ | ✓ | ✓ | ✓ | NA* | **×** | **×** | 9 (**64%**) |
| Roiger et al. [37] | ✓ | **×** | ✓ | NR | ✓ | ✓ | ✓ | ✓ | **×** | ✓ | **×** | ✓ | **×** | NA* | **×** | 8 (**57%**) |
| Singh et al. [38] | ✓ | ✓ | NR | NR | ✓ | ✓ | **×** | ✓ | ✓ | ✓ | NR | ✓ | **×** | ✓ | **×** | 9 (**60%**) |
| Strain et al. [39] | ✓ | ✓ | NR | NR | CD | ✓ | **×** | **×** | ✓ | ✓ | **×** | ✓ | NA* | NA* | **×** | 6 (**46%**) |
| Vargas et al. [40] | ✓ | ✓ | ✓ | NR | ✓ | **×** | **×** | ✓ | ✓ | ✓ | **×** | ✓ | NA* | NA* | ✓ | 9 (**69%**) |
| Yang et al. [41] | ✓ | **×** | ✓ | NR | ✓ | ✓ | **×** | ✓ | **×** | ✓ | **×** | ✓ | NA* | NA* | ✓ | 8 (**62%**) |

*Note.*^a^**=**same sample as Kerr et al. [23], ^b^ =same sample as Mainwaring et al. [27] MH=mental health**,** NR=not reported, NA=not applicable, CD=cannot determine, *denotes NA item not included as denominator**,** ^#^denominators vary where NA* items have been excluded
